# Comparison of image quality and acquistion time of Free-Breathing (FB) motion corrected (MOCO) SSFP to current PSIR sequences: is one sequence superior?

**DOI:** 10.1186/1532-429X-17-S1-P18

**Published:** 2015-02-03

**Authors:** Ryan Avery, Rajesh Janardhanan, Clinton Jokerst

**Affiliations:** 1 Medical Imaging, University of Arizona, Tucson, AZ, USA

## Background

Delayed myocardial enhancement (DE) sequences have become essential in cardiac MRI (CMRI) for visualization of abnormal myocardium. DE sequences require an inversion pulse to null normal myocardium, and the introduction of phase-sensitive inversion recovery (PSIR) sequences, which are less sensitive to small variations in inversion time (TI), have provided a suitable alternative to magnitude inversion recovery sequences. Two PSIR sequences are widely available, the single-shot SSFP PSIR (SS-SSFP) and segmented turbo FLASH (STF) PSIR sequences. The SS-SSFP PSIR sequence has a short acquisition time making it motion insensitive, but also limiting resolution. The STF PSIR is a longer sequence with better resolution, but also requires several breath holds. The recent introduction of a free-breathing (FB) motion-corrected (MOCO) version of the SS-SSFP PSIR (FB MOCO SSFP) may offer a compromise between image resolution and acquisition time by combining the speed and motion-insensitivity of SS-SSFP PSIR with a resolution similar to STF PSIR. Since CMRI is a time-intensive exam, comparison of these sequences regarding both image quality and acquisition time is necessary.

## Methods

13 patients (8F, 4M, age 22-76 years, mean 52) underwent DE sequences with a 1.5T MR scanner (Siemens MAGNETOM Aera, Erlangen, AG) 10-25 minutes after IV gadolinium administration. DE sequences included breath hold (BH) SS-SSFP, FB MOCO SSFP, and BH STF. Cardiomyopathy (n=6, 46.1%) and myocarditis (n=3, 23.1%) were the most common indications. Two experienced cardiovascular imagers graded each of the three PSIR sequences using a 5-point Likert scale regarding image motion degradation, image quality, DE identification, and overall diagnostic confidence. Left ventricular DE was quantified using a 16-segment model, and image acquisition time of each PSIR sequence was recorded.

## Results

Mean grading of BH SS-SSFP, FB MOCO SSFP, and BH STF was, respectively, for image motion degradation 4.9, 4.9, and 3.7; image quality 3.5, 4.7, and 3.9; and DE visualization 3.4, 4.8, and 3.5. DE was seen in 9 cases with a total of 39 segments involved. No discrepancy between segments involved was determined amongst sequences or readers. The mean overall diagnostic confidence in the PSIR sequences was 3.6, 4.5, and 3.5, respectively. The average acquisition times of the respective PSIR sequences were 48.5 +/- 4.3s, 78.8 +/- 27.9s, and 310.8 +/- 97.0s.

## Conclusions

FB MOCO SSFP demonstrated superior image quality, DE localization, and overall diagnostic confidence compared to BH SS-SSFP and BH STF PSIR sequences. Additionally, no discrepancies between reader or sequence were found during DE segmental identification. While further investigation is warranted, FB MOCO SSFP is a suitable alternative to the current PSIR sequences. Furthermore, when PSIR image acquisition times were compared, these findings suggest FB MOCO SSFP is markedly superior to BH STF, and a suitable replacement.

## Funding

Master Research Agreement with Siemens Healthcare.

